# Comparison of gene expression profiles in the blood, hippocampus and prefrontal cortex of rats

**DOI:** 10.1186/2193-9616-1-15

**Published:** 2013-11-13

**Authors:** Stephanie H Witt, Wolfgang H Sommer, Anita C Hansson, Carsten Sticht, Marcella Rietschel, Christian C Witt

**Affiliations:** Department of Genetic Epidemiology in Psychiatry, Central Institute of Mental Health, Medical Faculty Mannheim/Heidelberg University, J5, 68159 Mannheim, Germany; Institute of Psychopharmacology, Central Institute of Mental Health, Medical Faculty Mannheim/Heidelberg University, J5, 68159 Mannheim, Germany; Medical Research Center, University Hospital Mannheim, Medical Faculty Mannheim/Heidelberg University, Theodor-Kutzer-Ufer 1-3, 68167 Mannheim, Germany; Department of Anaesthesiology and Operative Intensive Care, University Hospital Mannheim, Medical Faculty Mannheim/Heidelberg University, Theodor-Kutzer-Ufer 1-3, 68167 Mannheim, Germany

**Keywords:** Gene expression, Brain, Blood, Comparison, Rat

## Abstract

**Background:**

The comparability of gene expression between blood and brain tissues is a central issue in neuropsychiatric research where the analysis of molecular mechanisms in the brain is of high importance for the understanding of the diseases and the discovery of biomarkers. However, the accessibility of brain tissue is limited. Therefore, knowledge about how easily accessible peripheral tissue, e. g. blood, is comparable to and reflects gene expression of brain regions will help to advance neuropsychiatric research.

**Description:**

Gene expression in the blood, hippocampus (HC) and prefrontal cortex (PFC) of genetically identical rats was compared using a genome-wide Affymetrix gene expression microarray covering 29,215 expressed genes. A total of 56.8% of 15,717 expressed genes were co-expressed in blood and at least one brain tissue, while 55.3% of all genes were co-expressed in all three tissues simultaneously. The overlapping genes included a set of genes of relevance to neuropsychiatric diseases, in particular bipolar disorder, schizophrenia and alcohol addiction. These genes included CLOCK, COMT, FAAH, NPY, NR3C1, NRGN, PBRM1, TCF4, and SYNE.

**Conclusions:**

This study provides baseline data on absolute gene expression and differences between gene expression in the blood, HC and PFC brain tissue of genetically identical rats. The present data represents a valuable resource for future studies as it might be used for first information on gene expression levels of genes of interest in blood and brain under baseline conditions. Limitations of our study comprise possible contamination of brain tissue with blood and the non-detection of genes with very low expression levels. Genes that are more highly expressed in the brain than in the blood are of particular interest since changes in their expression, e.g. due to disease status, or treatment, are likely to be detected in an experiment. In contrast, genes with higher expression in the blood than in the brain are less informative since their higher baseline levels could superimpose variation in brain.

## Background

The pathogenesis of neuropsychiatric diseases still remains poorly understood. Although genome-wide association studies have generated robust evidence for the involvement of various of genes in a number of psychiatric disorders (for a review see: Sullivan et al. 2012) the underlying molecular mechanisms are still await clarification (Schulze 2010). Identification of the biological factors that underlie these diseases is difficult since it requires observation of biological processes such as gene- and protein expression in tissues like the brain, which are difficult to access.

A useful approach to the identification of the molecular mechanisms implicated in the etiology and underlying processes of neuropsychiatric disease is to study genes that are differentially expressed during illness phases and well-being or between patients and healthy controls. This provides an important tool for discovering molecular mechanisms which are involved in disease etiology and disease processes. However, due to the inaccessibility of brain tissue, most gene expression studies involve either post-mortem brain samples or readily obtainable peripheral tissue, in particular blood. The question therefore arises, as to whether and to what extent gene expression in peripheral blood samples is comparable to gene expression in the brain.

To date, few studies have addressed these important questions. In 2006, Sullivan et al. compared transcripts in human blood and post-mortem brain and found a median non-parametric correlation between transcripts present in both whole blood and the central nervous system (CNS) of around 0.5. The authors suggested that the use of peripheral gene expression may be a useful surrogate for gene expression in the brain, provided that the genes of interest are definitely expressed in both types of tissue (Sullivan et al. 2006). However, this study involved random human blood and CNS samples. The heterogeneity of genetic background in humans and the general methodological problems associated with the use of post-mortem brain samples therefore limit the interpretability and applicability of these results. Sullivan et al. concluded that animal studies would be informative (Sullivan et al. 2006). Peripheral blood mononuclear cell (PBMC) profiles showed co-expression levels of summarized transcripts for 4,103 of 17,859 (22.9%) transcripts and rat hemi-brain and 19% of transcripts blood showed similar expression. However, at the time of writing, no comprehensive reference database for absolute gene expression levels in the blood and specific brain regions of animals is available.

In a study with vervet monkeys, gene expression profiles in eight brain tissues and blood of 12 vervet monkeys was analyzed (Jasinska et al. 2009). The focus of this study was to find high inter- and intraindividual brain gene expression differences displayed in the blood to identify candidate genes for mapping brain eQTL in peripheral blood. They find a co-expression of 2,430 genes over all tissues and blood in twelve animals. However, in comparison to age matched inbred rat strains, results are confounded by a high interindividual heterogeneity and more imprinting. Moreover, vervets are not a feasible animal model for challenging experiments. Challenging – e.g. by alcohol consumption – would be a necessary intervention to analyse how changes in brain gene expression are reflected in the blood.

In our study, we analyzed gene expression profiles of genetically identical rats in blood, hippocampus and prefrontal cortex in order to assess baseline values of concordant and differential gene expression between these tissues. We chose to collect these data in animal models to avoid confounding factors which usually limit the interpretability of data from humans.

## Construction and content

### Blood and tissue sampling, and RNA preparation

Four male 6-week old Wistar rats (Charles River, Sulzfeld, Germany), were housed for 1 week under standard conditions (2 per cage, 12-h artificial light–dark cycle, lights on 7:00, temperature: 22° ± 1°C, humidity: 55% ± 5%, food and water ad libitum). Animals were sacrificed by decapitation (around 10 am). Blood samples were immediately collected in PAXgene tubes containing a stabilizing agent for RNA (Preanalytix, BD) and rat brains were quickly removed, submerged for 3 minutes in −40°C isopentane (Sigma-Aldrich Co., St. Louis, Missouri). All samples were stored at −80°C. For dissection, brains were sliced in coronal sections of 120 μm in a Leica CM3000 Cryostat (Leica, Bensheim, Germany). PFC and HC tissues were punched out, collected into vials and stored at −80°C. For RNA isolation, punched tissue were lysed in TRIzol® Reagent (Invitrogen, Karlsruhe, Germany) and homogenized by passing the suspension 30 times through a 22-gauge needle. Total RNA was extracted by adding chloroform. To achieve better separation of organic and aqueous phases, Phase Lock Gel™ Heavy tubes (Eppendorf, Hamburg, Germany) were used. Upper phases were carefully removed by pipetting and total RNA was purified using RNeasy® Micro Kit (Qiagen, Hilden, Germany). RNA from blood samples was isolated with PAXgene Blood RNA Kit 50 (PreAnalytiX, Qiagen) according to the manufactures recommendation. Total RNA yield was determined with Quant-iT™ RiboGreen® RNA Reagent and Kit (Invitrogen) by measuring in a Wallac Victor 2 1420 Multilabel Counter (Perkin Elmer, Jügesheim, Germany). Total RNA purity was evaluated by OD measurements (260 nm/280 nm) in a NanoDrop (peqLab, Erlangen, Germany) and its integrity was determined by RNA integrity number (RIN) measurement using RNA 6000 Nano Assay RNA chips run in an Agilent 2100 Bioanalyzer (Agilent Technologies, Palo Alto, CA). Ratios of 1.9–2.2 (OD 260/280) and RIN > 8.0 as well as an absence of a peak of genomic DNA contamination in electropherograms were chosen as inclusion criteria.

### Microarry processing and statistical analysis

Gene expression profiling was performed using GeneChip Rat Gene 1.0 ST Array (Affymetrix, Inc., Santa Clara, CA, USA). Samples were transcribed to cDNA and hybridized to Affymetrix GeneChip Rat Gene 1.0 ST Array (Affymetrix, Inc., Santa Clara, CA) per the Whole Transcript (WT) Sense Target Labeling Assay protocol [Affymetrix 2006] using 200 ng of total RNA from each sample. The raw fluorescence intensity values were imported, quantile normalized and RMA transformed using the affymetrix power tools (apt-1.51). To define a transcript/gene as detected, the dabg (Detected Above Background) p-values were calculated with apt-program. It calculates the p-value that the intensities in a probeset could have observed by chance in a background distribution. When the dabg-p-value was smaller or equal than 0.05 and the mean of all three RMA-intensities were higher than 6.2 (the overall mean of all probeset-intensities), the probe set was defined as “present”. One sample did not pass the quality criteria and was omitted from the analysis.

### Analysis of genes implicated in psychiatric diseases

The gene expression data was analyzed with respect to the presence of a selection of genes that have been described to be relevant for psychiatric diseases, especially bipolar disorder, schizophrenia and alcohol addiction (Chen et al. 2013; Green et al. 2013; Hodgkinson et al. 2008; Lee et al. 2012; McMahon et al. 2010; Mitchell and Porteous 2011; Schizophrenia Psychiatric Genome-Wide Association Study C 2011; Shi et al. 2011; Stefansson et al. 2009; Steinberg et al. 2011; Vassos et al. 2012).

### Absolute gene expression in blood, PFC and HC

**Figure 1:**
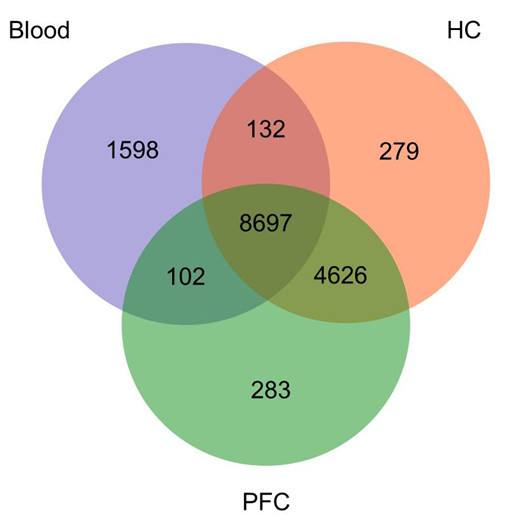
**Venn diagramm: absolute number of expressed genes found in blood, PFC and HC.**

Figure 1 (Venn diagram) shows the absolute number of expressed genes found in blood, PFC and HC. Table 1 depicts number and percentages of total expressed genes and of the overlap in the different tissues (cut-off level of 6.2 for all data and present in all samples p < 0.05). All raw and normalized data have been deposited in the Gene-expression Omnibus database (http://www.ncbi.nlm.nih.gov/geo/; accession number GSE49352).

**Table 1:** **Number and percentage of transcript in different tissues and overlap**

	Total number	Percentage of all expressed genes	Percentage of genes expressed in blood	Percentage of genes restricted to respective tissue*
**All expressed genes**	15,717	100		
**Blood all**	10,529	67.0	100	
**Blood only**	1,598	10.2	-	15.2
**Brain all**	14,119	89.8		
**PFC all**	13,708	87.2	-	
**PFC only**	283	1.8	-	2.1
**HC all**	13,734	87.4	-	
**HC only**	279	1.8	-	2.0
**Brain only**	5,188	33.0	-	36.8
**Overlap blood, brain**	8,931	56.8	84.8	
**Overlap blood & PFC & HC**	8,697	55.3	82.6	
**Overlap blood & PFC only**	102	0.7	1.0	
**Overlap blood & HC only**	132	0.8	1.3	
**Overlap PFC & HC only**	4,626	29.4		

*of all genes expressed in blood or brain respectively.

Of the 29,215 probe sets from the Rat Gene 1.0 ST Array read into jmp genomics, the total number and percentage of expressed genes at a cut-off level of 6.2 in all investigated tissues (blood, PFC and HC) is 15,717 (53.8%). Of these genes, 10,529 (67.0%) are expressed in blood, 13.708 (87.2%) in PFC, and 13,734 (87.4%) in HC respectively.

Of all genes that are expressed in the blood, 8,931 (84.8%) are also expressed in at least one brain tissue (Table 1 and Figure 1). 8,697 (82.6%) of blood-expressed genes are expressed in both PFC and HC.

1,598 (10.2%) of the detected mRNAs are detected exclusively in the blood, whereas 4,626 (29.4%) transcripts are only detectable in both PFC and HC. Another 562 (3.6%) genes are expressed in either PFC or HC only adding up to a total number of 5,188 (33.0%) of exclusively brain-expressed genes.

### Comparison of genes implicated in psychiatric diseases

**Figure 2:**
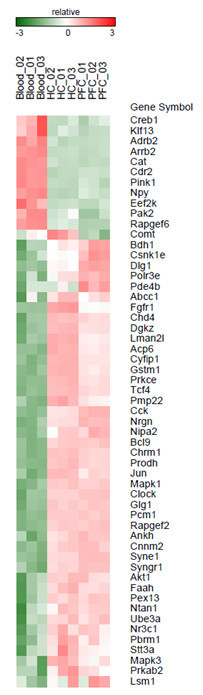
**Selection of formerly described bipolar disorder, schizophrenia and alcohol addiction genes, which are present as expressed transcripts according to more than one tissue type.** Depicted are absolute expression levels of individual animals.

The comparison of genes implicated in psychiatric disorders revealed a set of genes that is co-expressed in blood, PFC and HC (Figure 2).

## Utility

The issue of comparability of gene expression in the blood and the brain is crucial for studies of psychiatric disorders where access to brain tissue is limited. In using blood as a substitute for brain tissue, knowledge of both the degree of overlap in and the tissue-specificity of gene expression is crucial.

The present study provides a baseline data concerning absolute gene expression and differences in gene expression between the blood, PFC and HC brain tissue in genetically identical rats. The present data represents a valuable resource for future studies as it might be used for information on gene expression levels of genes of interest in blood and brain under baseline conditions.

A large number of genes were co-expressed in blood and brain tissue, and approximately 60% of genes blood-expressed were also present in at least one brain region. Congruent with the results of Jasinska et al. (2009), in the present study, housekeeping genes presented a large portion of overlapping expressed genes. For 59% of co-expressed genes, gene expression was higher in the brain than in the blood. This group of genes is especially informative since changes in their expression, e.g. due to disease status, or treatment, are likely to be detected in an experiment. In contrast, genes with higher expression in the blood than in the brain are less informative since their higher baseline levels could superimpose variation in brain.

As expected, a number of genes were co-expressed in blood and one brain region only. Information on co-expressed genes that are specific to blood and a single brain region allows to relate blood gene expression changes to different regions in the brain.

The brain regions analyzed in the present study, i.e. the PFC and HC - are very different in terms of both, function and structure. Nevertheless, a large number of genes were expressed in both tissues and only a small subset that is specific for a single brain region. Although the group of brain region specific genes is small, research has shown that differences between brain regions are the most important source of variance in quantitative assessments of expression profiles (Matthaus et al. 2009; Reimers et al. 2005). Genes with overlapping expression in blood and brain included a set of genes which of relevance for psychiatric diseases, in particular bipolar disorder, schizophrenia and alcohol addiction (Chen et al. 2013; Green et al. 2013; Hodgkinson et al. 2008; Lee et al. 2012; McMahon et al. 2010; Mitchell and Porteous 2011; Schizophrenia Psychiatric Genome-Wide Association Study C 2011; Shi et al. 2011; Stefansson et al. 2009; Steinberg et al. 2011; Vassos et al. 2012). These include CLOCK, COMT, FAAH, NPY, NR3C1, NRGN, PBRM1, TCF4, and SYNE (Figure 2).

## Discussion

The aim of this study was to compare blood and brain gene expression levels in the same animal at the same time point and in order to confounders attributable to the collection of biomaterial. To optimize this approach, genetically identical rats were chosen for analysis. The advantage of this approach is that it ensured a high degree of homogeneity in terms environmental conditions. A shortcoming of the approach is that differences in gene expression exist in rats compared to humans. Moreover, the low number of samples precluded any analysis of quantitative differences.

A promising strategy for future studies, it would be desirable to be able to compare blood and brain gene expression in humans, e.g. of material which is collected during brain surgery. Even during scrupulous biomaterial collection, the possibility of contamination of brain tissue with blood cannot be entirely exluded. Therefore, some of the genes co-expressed in blood and brain are due to this contamination. However, blood contamination should lead to only very low levels of gene expression that should lie below the applied cut-off level. Moreover, there is a number of genes exclusively expressed in the brain (33.0%, Table 1) suggesting that the level of contamination of the brain expression profiles by blood specific transcripts was low.

Gene expression varies widely across different brain regions (Hansson et al. 2006). Therefore, in future studies, analysis of the co-expression of genes in blood and brain regions other than the HC and PCF would be of interest.

Furthermore, since miRNAs are also of interest in psychiatric research, data concerning the comparability of miRNAs levels in the blood and in brain would be desirable (Tapocik et al. 2013).

The present analysis generated data on baseline gene expression levels in wild-type rats. Gene expression analyses in the blood and brain of rats which have undergone some form of challenge or which differ in disease state would also be of interest since they would facilitate the elucidation of if and how changes of brain gene expression might be reflected in blood.

## Conclusion

Knowledge of the comparability of gene expression in blood and brain is an important issue in neuropsychiatric research. Although further aspects need to be addressed, the present study is the first to provide a systematic analysis of absolute gene expression levels in blood and single brain regions. Genes higher expressed in the brain than in blood are of particular interest since changes in gene expression in this readily accessible tissue may reflect disease status, or treatment.
